# Development and validation of a risk prediction model for end-stage renal disease in patients with type 2 diabetes

**DOI:** 10.1038/s41598-017-09243-9

**Published:** 2017-08-31

**Authors:** Cheng-Chieh Lin, Chia-Ing Li, Chiu-Shong Liu, Wen-Yuan Lin, Chih-Hsueh Lin, Sing-Yu Yang, Tsai-Chung Li

**Affiliations:** 1 School of Medicine, College of Medicine, China Medical University, Taichung, Taiwan; 2 Department of Family Medicine, China Medical University Hospital, Taichung, Taiwan; 3 Department of Medical Research, China Medical University Hospital, Taichung, Taiwan; 4 Department of Public Health, College of Public Health, China Medical University, Taichung, Taiwan; 5 Department of Healthcare Administration, College of Medical and Health Science, Asia University, Taichung, Taiwan

## Abstract

The aim of this study is to develop a prediction model for ESRD in patients with type 2 diabetes. A retrospective cohort study was conducted, consisting of 24,104 Chinese patients with type 2 diabetes. We adopted the procedures proposed by the Framingham Heart Study to develop a prediction model for ESRD. Participants were randomly assigned to the derivation and validation sets at a 2:1 ratio. The Cox proportional hazard regression model was used for model development. A total of 813 and 402 subjects (5.06% and 5.00%, respectively) developed ESRD in the derivation and validation sets over a mean follow-up period of 8.3 years. The risk-scoring systems included age, gender, age of diabetes onset, combined statuses of blood pressure and anti-hypertensive medication use, creatinine, variation in HbA1c, variation in systolic blood pressure, diabetes retinopathy, albuminuria, anti-diabetes medications, and combined statuses of hyperlipidemia and anti-hyperlipidemia medication use. The area under curves of 3-year, 5-year, and 8-year ESRD risks were 0.90, 0.86, and 0.81 in the derivation set, respectively. This risk score model can be used as screening for early prevention. The risk prediction for 3-year, 5-year, and 8-year period demonstrated good predictive accuracy and discriminatory ability.

## Introduction

Based on WHO report, the standardized mortality rate of diabetes increased from 23.6/10^5^ in 1985 to 39.4/10^5^ in 2006. This increase made diabetes the most rapidly increasing ailment compared with other chronic diseases. Diabetes and its complications are the leading causes of premature mortality and impose a heavy burden on the individual and societal levels^1–3^. Diabetes is also a leading cause of end-stage renal disease (ESRD) in many developed and developing countries^4^; about 44.6%, 44.5%, and 43.7% ESRD incidence in patients in Japan, Taiwan, and the United States, respectively, are caused by diabetes^5^. Given the alarming rise in the number of diabetes cases worldwide^6^, the ESRD population is increasing. In Taiwan, the prevalence and incidence of ESRD is increasing rapidly^7^. The total number of regular dialysis patients increased by 26.5% from 52 081 in 2006, to 65 883 in 2010^8^. The rising number of ESRD patients who require dialysis therapy or transplantation is a major public health problem; such problem places a substantial burden on affected individuals and health care system^5^.

Predicting ESRD risks in patients with type 2 diabetes can help guide individualized interventions for secondary prevention and future health care needs. The prediction models or point systems quantify the impact of measurable and modifiable risk factors. The advantage of these point systems incorporated complex statistical models that allowed health professionals and practitioners to use in clinic settings. Patients can easily estimate their own disease risk and monitor their risk over time. These point systems can also be used as a tool for clinicians to guide their decision making regarding treatment and for patients to promote motivation on modifying their behavior. Previous studies established prediction models for various kidney diseases, including CKD or ESRD in general population^9^, in patients with CKD^10–12^, and in patients with type 2 diabetes and CKD^13–15^. Among those studies targeting at patients with type 2 diabetes, two of them specified their population as those with diabetic nephropathy. One study targeting participants with type 2 diabetes without diabetic nephropathy focused on the composite outcome of major kidney-related event, including doubling of serum creatinine to 2.26 mg/dl, renal replacement therapy, or renal death^15^. Composite measure has the advantage of being more powerful due to low incidence rates of events, but it has the disadvantage of being less specific. Because the prevention strategy for kidney disease, ESRD, or renal death was different, there is a need to develop prediction model for ESRD. No prior model has been developed for patients with type 2 diabetes on ESRD outcome or in the Chinese population. In addition, prior studies reported that visit-to-visit variation in plasma glucose and blood pressure were associated with ESRD and these two factors haven’t been considered in prediction models of ESRD^16, 17^. This deficiency results in a need to construct a prediction model of ESRD risk in Chinese patients with type 2 diabetes. With a large cohort of 24,104 participants enrolled from clinical centers in the entire country, the Taiwan Diabetes Study (TDS) cohort represents a significant opportunity to examine whether the characteristics of patients at baseline were associated with ESRD incidence. This prediction model considered risk factors that are generally available in clinical practice and are precisely measured to ensure that this score system is acceptable in clinical practice. In the present study, we develop a point-based risk prediction model for incident ESRD using TDS data.

## Results

Taiwan Diabetes study included 24,104 type 2 diabetic patients aged 30–84 years old. These patients were free of ESRD at baseline and had been followed-up for 8.3 years. We randomly assigned 16,070 patients into the derivation set and 8,034 patients into the validation set. A total of 813 (5.06%) and 402 (5.00%) ESRD incident cases were found in the derivation and validation sets, respectively. Table 1 shows the baseline characteristics of participants according to derivation and validation sets. The mean age of participants in the derivation and validation sets was both about 61 years; 47% of the samples were male. All baseline socio-demographic, clinical, and laboratory variables had standardized effect size values less than 0.1, demonstrating that variables in the two sets were distributed similarly.

**Table 1:** Baseline characteristic of the study population in derivation and validations.

	Derivation Set (n = 16,070)	Validation Set (n = 8,034)	Standardized effect size
	MEAN ± SD* or n (%)	MEAN ± SD* or n (%)	
*Socio-demographic factors*			
Age (years)	61.03 ± 10.77	61.19 ± 10.75	−0.01
Gender			−0.01
Female	8508 (52.94)	4307 (53.61)	
Male	7562 (47.06)	3727 (46.39)	
Smoking habit	2526 (15.72)	1251 (15.57)	0.00
Alcohol drinking	1476 (9.18)	710 (8.84)	0.01
Age of diabetes onset (years)	54.41 ± 10.88	54.51 ± 10.72	−0.01
Duration of type 2 diabetes (years)	6.66 ± 6.34	6.72 ± 6.36	−0.01
Body mass index (kg/m^2^)	25.68 ± 3.74	25.76 ± 3.76	−0.02
Obesity	5861 (36.47)	2993 (37.25)	−0.02
*Diabetes-related factor and biomarker*			
HbA1c level (%)	8.18 ± 1.92	8.24 ± 1.95	−0.03
Fasting blood glucose (mg/dL)	171.62 ± 65.39	171.77 ± 63.69	0.00
Low-density lipoprotein (mg/dL)	118.00 ± 31.11	117.58 ± 31.58	0.01
High-density lipoprotein (mg/dL)	46.19 ± 13.97	46.44 ± 14.04	−0.02
Creatinine (mg/dL)	1.05 ± 0.54	1.05 ± 0.52	0.00
Serum glutamate-pyruvate transaminase (u/l)	32.02 ± 31.54	32.52 ± 34.99	−0.02
Total cholesterol (mg/dL)	195.54 ± 41.64	196.09 ± 42.82	−0.01
Triglyceride (mg/dL)	170.42 ± 128.74	174.91 ± 135.13	−0.03
eGFR (mg/dL)	73.48 ± 21.60	73.17 ± 22.13	0.01
Variation in fasting blood glucose (%)	31.98 ± 25.62	32.07 ± 25.76	0.00
Variation in HBA1c (%)	16.51 ± 14.74	16.4 ± 14.45	0.01
*Comorbidity*			
Systolic blood pressure (mm Hg)	134.8 ± 17.71	134.97 ± 17.73	−0.01
Diastolic blood pressure (mm Hg)	80.05 ± 10.48	80.03 ± 10.54	0.00
Variation in systolic blood pressure (%)	7.37 ± 5.52	7.43 ± 5.63	−0.01
Variation in diastolic blood pressure (%)	7.67 ± 6.10	7.78 ± 6.10	−0.02
Hypertension	7218 (44.92)	3813 (47.46)	−0.05
Stroke	790 (4.92)	386 (4.8)	0.01
Cardiovascular disease	1402 (8.72)	748 (9.31)	−0.02
Peripheral Neuropathy	147 (0.91)	73 (0.91)	0.00
Diabetes retinopathy	216 (1.34)	108 (1.34)	0.00
Hypoglycemia	666 (4.14)	350 (4.36)	−0.01
Chronic kidney disease	155 (0.96)	77 (0.96)	0.00
Ketoacidosis	130 (0.81)	66 (0.82)	0.00
Postural hypotension	14 (0.09)	7 (0.09)	0.00
Arterial embolism and thrombosis	23 (0.14)	15 (0.19)	−0.01
Peripheral vascular disease	132 (0.82)	76 (0.95)	−0.01
Hyperlipidemia	4256 (26.48)	2138 (26.61)	0.00
Albuminuria	1890 (11.76)	1003 (12.48)	−0.02
*Medication use*			
Anti-diabetes medications			
No medication	635 (3.95)	309 (3.85)	0.01
Oral only	13823 (86.02)	6899 (85.87)	0.00
Insulin	616 (3.83)	287 (3.57)	0.01
Insulin + oral agent	996 (6.2)	539 (6.71)	−0.02
Hypertension medications	8287 (51.57)	4257 (52.99)	−0.03
Cardiovascular medications	5232 (32.56)	2608 (32.46)	0.00
Hyperlipidemia medications	6420 (39.95)	3234 (40.25)	−0.01
NSAID	143 (0.89)	79 (0.98)	−0.01
*Outcome*			
ESRD	813 (5.06)	402 (5.00)	0.00

^*^SD = standard deviation.

In crude Cox’s proportional hazard analysis, significant factors were observed for age groups of 60–64 years (4.05, 1.01–16.29), 75–79 (4.61, 1.14–18.66) years, and 80–84 years (4.50, 1.07–18.93); age of diabetes onset ≥45 years (0.66, 0.58–0.75), creatinine levels of 2.0–4.0 mg/dL (24.54, 21.25–28.34) and ≥4.0 mg/dL (19.01, 13.85–26.09), the second tertile of HbA1c-CV (1.45, 1.26–1.68), the third tertile of HbA1c-CV (1.70, 1.47–1.96), the third tertile of SBP-CV (1.48, 1.29–1.69), diabetes retinopathy (4.55, 3.51–5.88), albuminuria (1.91, 1.66–2.20), anti-diabetes medication of insulin monotherapy (5.16, 3.63–7.35) and insulin plus OAD (3.02, 2.13–4.28), combined statuses for blood pressure and anti-hypertensive medication: no anti-hypertensive medication and SBP 140–159 mmHg or DBP 90–99 mmHg (1.76, 1.30, 2.38), no anti-hypertensive medication and SBP ≥160 mmHg or DBP ≥ 100 mmHg (2.39, 1.57–3.65), anti-hypertensive medication use and SBP < 130 mmHg and DBP < 85 mmHg (3.39, 2.59–4.43), anti-hypertensive medication use and SBP 130–139 mmHg or DBP 85–89 mmHg (3.91, 3.03–5.04), anti-hypertensive medication use and SBP 140–159 mmHg or DBP 90–99 mmHg (4.77, 3.76, 6.06), anti-hypertensive medication use and SBP ≥160 mmHg or DBP ≥ 100 mmHg (6.86, 5.34, 8.83), and combined statuses of hyperlipidemia and anti-hyperlipidemia medication: no hyperlipidemia and total cholesterol 200–239 mg/dl (1.41, 1.16–1.70), no hyperlipidemia and total cholesterol ≥240 mg/dl (2.40, 1.85–3.10), anti-hyperlipidemia medication use and total cholesterol <200 mg/dl (1.93, 1.63–2.29), anti-hyperlipidemia medication use and total cholesterol 200–239 mg/dl (1.91, 1.60–2.28), and anti-hyperlipidemia medication use and total cholesterol ≥240 mg/dl (3.62, 3.05–4.29) (all p-value < 0.05) (Table 2).

**Table 2:** Number of participants, numbers and incidence rates of ESRD, and crude hazard ratios by baseline ESRD predictors.

Variables	N	Cases	Person-years	Incidence Rate	Crude HR (95% CI)	P value
*Socio-demographic factors*						
Age (years)						
30–34	133	2	1177	1.70	1.00	
35–39	474	5	4246	1.18	0.69 (0.13, 3.55)	0.66
40–44	1146	37	10136	3.65	2.14 (0.52, 8.89)	0.29
45–49	2037	72	17898	4.02	2.37 (0.58, 9.65)	0.23
50–54	3216	179	27923	6.41	3.78 (0.94, 15.24)	0.06
55–59	3192	169	27315	6.19	3.65 (0.90, 14.7)	0.07
60–64	4052	236	34158	6.91	4.05 (1.01, 16.29)	0.05
65–69	3876	212	31760	6.68	3.95 (0.98, 15.90)	0.05
70–74	3426	173	26540	6.52	3.97 (0.99, 16.02)	0.05
75–79	1932	103	14026	7.34	4.61 (1.14, 18.66)	0.03
80–84	620	27	3936	6.86	4.50 (1.07, 18.93)	0.04
Gender						
Female	12815	635	107541	5.90	1.00	
Male	11289	580	91574	6.33	1.09 (0.97, 1.22)	0.14
Age of diabetes onset (years)						
<45	4773	340	40570	8.38	1.00	
≥45	19331	875	158545	5.52	0.66 (0.58, 0.75)	<0.001
*Diabetes-related factor and biomarker*						
Creatinine (mg/dL)						
≤2.0	23517	937	196164	4.78	1.00	
2.0–4.0	498	238	2479	96.01	24.54 (21.25, 28.34)	<0.001
>4.0	89	40	472	84.75	19.01 (13.85, 26.09)	<0.001
Variation in HbA1c (%)						
<17.3	7828	324	65277	4.96	1.00	
17.3–34.5	8025	381	66902	5.69	1.45 (1.26, 1.68)	<0.001
>34.5	8251	510	66936	7.62	1.70 (1.47, 1.96)	<0.001
Variation in systolic blood pressure (%)						
<4.4	7948	349	65947	5.29	1.00	
4.4–8.7	7958	357	66634	5.36	1.02 (0.88, 1.19)	0.75
>8.7	8198	509	66535	7.65	1.48 (1.29, 1.69)	<0.001
*Baseline cardiovascular diseases*						
Diabetes retinopathy						
No	23780	1154	196721	5.87	1.00	
Yes	324	61	2394	25.48	4.55 (3.51, 5.88)	<0.001
Albuminuria						
No	21211	974	176107	5.53	1.00	
Yes	2893	241	23008	10.47	1.91 (1.66, 2.20)	<0.001
*Medication use*						
Anti-diabetes medications						
No medication	944	38	7476	5.08	1.00	
Oral only	20722	829	173596	4.78	0.91 (0.65, 1.25)	0.55
Insulin	903	165	6244	26.43	5.16 (3.63, 7.35)	<0.001
Insulin+oral agent	1535	183	11799	15.51	3.02 (2.13, 4.28)	<0.001
Anti-hypertensive medications						
No						
SBP < 130 and DBP < 85	4661	83	39929	2.08	1.00	
SBP:130–139 or DBP:85–89	3269	65	28074	2.32	1.10 (0.79, 1.52)	0.57
SBP:140–159 or DBP:90–99	2904	88	24294	3.62	1.76 (1.30, 2.38)	<0.001
SBP≥160 or DBP≥100	726	29	5933	4.89	2.39 (1.57, 3.65)	<0.001
Yes						
SBP < 130 and DBP < 85	2735	154	22226	6.93	3.39 (2.59, 4.43)	<0.001
SBP:130–139 or DBP:85–89	3125	211	25557	8.26	3.91 (3.03, 5.04)	<0.001
SBP:140–159 or DBP:90–99	4605	359	36922	9.72	4.77 (3.76, 6.06)	<0.001
SBP≥160 or DBP≥100	2079	226	16180	13.97	6.86 (5.34, 8.83)	<0.001
Anti-hyperlipidemia medication						
No						
Total cholesterol: <200	9706	299	80498	3.71	1.00	
Total cholesterol: 200–239	3738	163	31111	5.24	1.41 (1.16, 1.70)	<0.001
Total cholesterol: ≥240	1006	72	8112	8.88	2.40 (1.85, 3.10)	<0.001
Yes						
Total cholesterol: <200	3980	239	32698	7.31	1.93 (1.63, 2.29)	<0.001
Total cholesterol: 200–239	3493	208	29202	7.12	1.91 (1.60, 2.28)	<0.001
Total cholesterol: ≥240	2181	234	17496	13.37	3.62 (3.05, 4.29)	<0.001

Incidence rate = number of incident cases / person-years*1000; HR: hazard ratio; CI: confidence intervals.

Table 3 shows the adjusted regression coefficients, and means or proportions of significant risk factors that remained in the final multivariate Cox’s proportional hazards model. ESRD risk score of each factor was defined as 5 times the regression coefficient of age. The total risk score ranged from −16 to 97. The 3-, 5-, and 8-year risks of ESRD were estimated for total scores followed by the algorithm developed in the Framingham heart study (Table 4).

**Table 3:** ESRD risk score based on the final multivariate Cox’s proportional hazards model.

Risk factor	β (SE)	Mean or proportion	P-value	Risk score
*Socio-demographic factors*				
Age	0.02 (0.004)	61.03	<0.001	−2 to 8
Gender (male)	−0.01 (0.07)	0.47	0.89	0
Age of diabetes onset (years) (ref: <45)				
≥45	−0.56 (0.10)	0.80	<0.001	−6
*Diabetes-related factor and biomarker*				
Creatinine (mg/dL) (ref: <2.0)				
2.0–4.0	2.57 (0.10)	0.02	<0.001	29
>4.0	2.65 (0.22)	0.004	<0.001	30
Variation in HbA1c (%) (ref: <8.5)				
8.5–17.5	0.23 (0.09)	0.33	0.01	3
>17.5	0.48 (0.09)	0.34	<0.001	6
Variation in systolic blood pressure (%) (ref: <4.4)				
4.4–8.7	−0.08 (0.09)	0.33	0.40	−1
>8.7	0.24 (0.09)	0.34	0.005	3
*Diabetes-related disorders*				
Diabetes retinopathy	0.88 (0.17)	0.01	<0.001	10
Albuminuria	0.50 (0.09)	0.12	<0.001	6
*Medication use*				
Anti-diabetes medications (ref: no medication)	ref	ref	ref	0
Oral only	−0.60 (0.21)	0.86	0.004	−7
Insulin	0.45 (0.23)	0.04	0.05	5
Insulin+oral agent	0.12 (0.23)	0.06	0.60	2
Hypertension medications				
No				
SBP<130 and DBP<85	ref	ref	ref	0
SBP:130–139 or DBP:85–89	0.12 (0.21)	0.14	0.55	1
SBP:140–159 or DBP:90–99	0.46 (0.20)	0.12	0.02	5
SBP≥160 or DBP≥100	0.72 (0.25)	0.03	0.005	8
Yes				
SBP<130 and DBP<85	0.99 (0.17)	0.11	<0.001	11
SBP:130–139 or DBP:85–89	1.06 (0.17)	0.13	<0.001	12
SBP:140–159 or DBP:90–99	1.33 (0.16)	0.19	<0.001	15
SBP≥160 or DBP≥100	1.65 (0.17)	0.08	<0.001	19
Hyperlipidemia medications				
No				
Total cholesterol: <200	ref	ref	ref	0
Total cholesterol: 200–239	0.34 (0.12)	0.16	0.005	4
Total cholesterol: ≥240	0.53 (0.17)	0.04	0.002	6
Yes				
Total cholesterol: <200	0.36 (0.11)	0.17	<0.001	4
Total cholesterol: 200–239	0.35 (0.11)	0.15	0.002	4
Total cholesterol: ≥240	0.91 (0.11)	0.09	<0.001	10

**Table 4:** 3-, 5-, 8-year estimated risk for ESRD of each possible sum of points.

Point total	Predicted risk of ESRD		
	3-year risk, %	5-year risk, %	8-year risk, %
−16~−14	0.12~0.15	0.25~0.30	0.53~0.63
−13~−11	0.16~0.19	0.33~0.39	0.69~0.82
−10~−8	0.21~0.25	0.43~0.51	0.90~1.07
−7~−5	0.27~0.32	0.56~0.66	1.17~1.39
−4~−2	0.35~0.42	0.72~0.86	1.51~1.80
−1~1	0.46~0.54	0.94~1.12	1.96~2.33
2~4	0.59~0.71	1.22~1.45	2.54~3.01
5~7	0.77~0.92	1.58~1.88	3.28~3.90
8~10	1.00~1.19	2.05~2.44	4.25~5.04
11~13	1.30~1.54	2.65~3.15	5.48~6.49
14~16	1.68~2.00	3.43~4.08	7.06~8.35
17~19	2.18~2.59	4.44~5.26	9.08~10.71
20~22	2.82~3.35	5.73~6.78	11.63~13.69
23~25	3.65~4.33	7.38~8.72	14.84~17.41
26~28	4.72~5.59	9.48~11.18	18.83~22.00
29~31	6.09~7.20	12.13~14.28	23.75~27.59
32~34	7.84~9.26	15.47~18.14	29.69~34.25
35~37	10.06~11.86	19.62~22.89	36.72~42.01
38~39	12.87~13.95	24.70~26.62	44.82~47.73
40~41	15.12~16.38	28.66~30.83	50.73~5381
42~43	17.74~19.19	33.11~35.52	56.95~60.14
44~45	20.74~22.40	38.05~40.70	63.34~66.55
46~47	24.18~26.07	43.46~46.32	69.73~72.85
48~49	28.08~30.20	49.28~52.32	75.89~78.83
50~51	32.46~34.83	55.44~58.60	81.62~84.25
52~53	37.32~39.94	61.80~65.01	86.69~88.93
54~55	42.67~45.50	68.20~71.36	90.94~92.72
56~57	48.44~51.46	74.44~77.43	94.27~95.58
58~59	54.55~57.70	80.30~83.01	96.68~97.56
60~61	60.90~64.10	85.54~87.88	98.26~98.80
62~63	67.31~70.47	90.00~91.90	99.20~99.48
64~65	73.58~76.60	93.55~94.98	99.68~99.81
66~67	79.50~82.26	96.18~97.16	99.89~99.94
68~69	84.85~87.24	97.95~98.56	99.97~99.99
70~71	89.43~91.38	99.02~99.36	99.99~100.00
72~73	93.11~94.60	99.60~99.76	100.00
74~76	95.86~97.75	99.86~99.96	100.00
77~79	98.40~99.28	99.98~100.00	100.00
≥80	99.54~100.00	100.00	100.00

**Figure 1:**
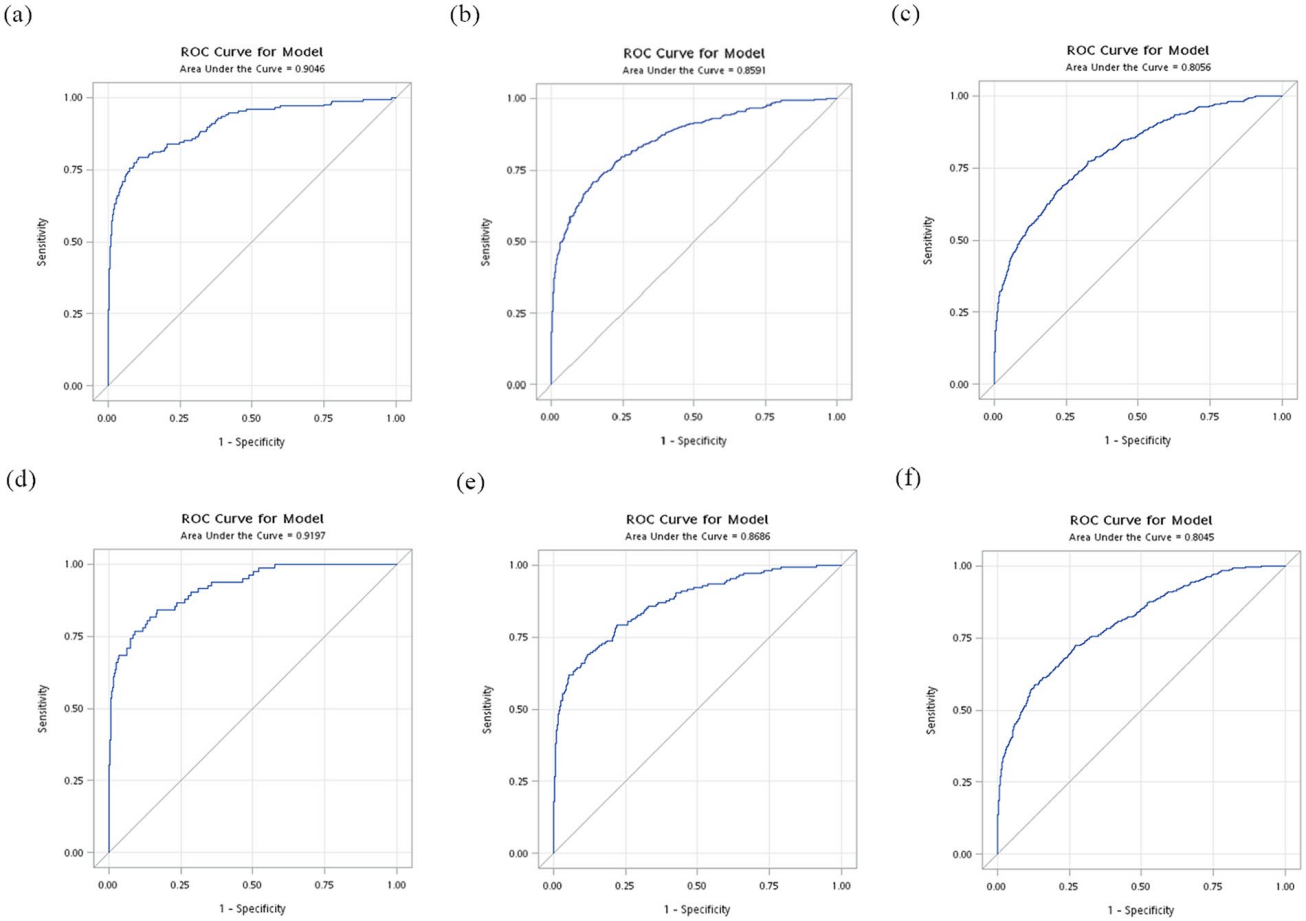
Receiver-operating characteristic curve (ROC) for (**a**) 3-year (**b**) 5-year (**c**) 8-year ESRD risk in derivation set and for (**d**) 3-year (**e**) 5-year (**f**) 8-year ESRD risk in validation set.

The areas under receiver operating curves (AUCs) of the final prediction model for 3-, 5-, and 8-year ESRD risks were 0.91, 0.86, and 0.81, respectively in the derivation set and were 0.92, 0.87, and 0.80, respectively in the validation set (Fig. 1). Our prediction model demonstrated excellent discrimination ability (i.e., AUCs ≥ 0.80) both in the derivation and validation sets.

**Figure 2:**
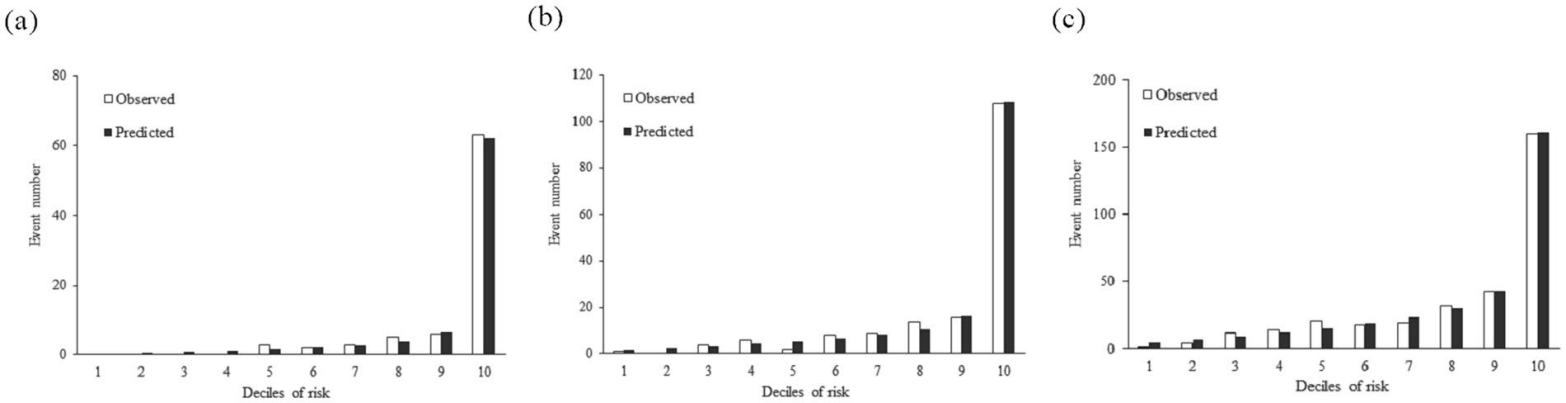
Predicted versus observed ESRD numbers according to deciles of (**a**) 3-year (**b**) 5-year and (**c**) 8-year risk in the validation set.

Figure 2 shows the calibration plots, showing the actual and predicted ESRD events according to deciles of 3-, 5-, and 8-year risks in the validation set. The ESRD events increased steadily from the sixth to 9^th^ deciles and then increased sharply at 10^th^ decile. The results of Hosmer–Lemeshow *x* ^2^ tests for 3-, 5-, and 8-year risk revealed no significant differences between the observed and predicted ESRD events in the validation set (all p > 0.05). The internal validation of the present model performance was assessed based on 1000 samples from bootstrap resampling. The optimism corrected calibration intercept was 0.009 with a mean absolute error of 0.00008 and the corresponding slope was 0.92 with a mean absolute error of 0.00191. These statistics indicate pretty good calibration for the present model and there is no need for shrinkage of regression coefficients in the prediction model.

We use multiple imputation method to handle missing data in HbA1c-CV and SBP-CV based on information on age, age of diabetes onset, and duration of type 2 diabetes in the sensitivity analysis. A total of 52,528 patients were included for this sensitivity analysis. The adjusted regression coefficients estimated from dataset with multiple imputation were similar to those from dataset with complete dataset (Supplementary Table 2). We used our risk score to predict ESRD risk for this imputed dataset. We found that the AUC values were 0.90 (95% CI: 0.88−0.91), 0.84 (0.83–0.85), and 0.79 (0.78–0.80) for 3-, 5-, and 8-year periods, respectively. These results were similar to those in our original risk model. Our risk prediction model was robust and not sensitive to missing data.

## Discussion

Our study established an ESRD risk prediction model in a nationwide cohort of patients with type 2 diabetes. We identified independent factors, including age, age of diabetes onset, combined statuses of blood pressure and anti-hypertensive medication, creatinine, HbA1c-CV, SBP-CV, diabetes retinopathy, albuminuria, anti-diabetes medication, and combined statuses of hyperlipidemia and antihyperlipidemia medications. The dominance of creatinine, diabetic retinopathy, combined statuses of blood pressure and antihypertensive medication, combined statuses of hyperlipidemia and anti-hyperlipidemia medication, and age in predicting risk is evident. The subsequent factors in terms of dominance were age at diabetes onset, albuminuria, and the clinical management of diabetes (such as HbA1c-CV) and blood pressure (such as SBP-CV). These predictors reflect both the intensity and duration of pathology associated with diabetes and its vascular complications. Utilizing a nationwide cohort from all clinical settings and backgrounds enabled us to confirm the importance of eGFR, albuminuria, other glucose and blood pressure variation indicator and complications in predicting the risk of ESRD events.

Creatinine is a clinical measurement of renal function and albuminuria is a measure of kidney damage. They are also major factors in our prediction model. Several studies have reported that serum albumin, serum creatinine, and eGFR are strongly associated with ESRD^10–14, 18–28^. Most of the prediction models for ESRD included these important factors; these factors were considered essential in predicting ESRD^10–14, 24, 28^. In addition to the assessment of markers of renal function, our model examined the contribution of socio-demographic factors, laboratory variables, diabetes-related factors, and medication use to increase prediction ability.

HbA1c and BP measurements were performed routinely for diabetes care. HbA1c reflects the average of blood glucose levels, but not glucose fluctuations. Visit-to-visit variation in HbA1c measured by HbA1c-CV could provide long-term fluctuation of blood glucose. Monitoring HbA1c during follow-up visits facilitated the availability of this glucose variation measure. Prior studies demonstrated that glucose variation is an independent predictor of ESRD^16, 29^. High BP may increase pressure on the kidney, which may cause kidney damage. Existing literature reports that poor blood pressure control is associated with increased ESRD risk^30, 31^. Similar results are observed in our study. We found that variation in HbA1c and SBP, and baseline BP are strong predictors of ESRD risk. Both variation in HbA1c and SBP were the novel predictors that were first considered in the risk score prediction model.

Most studies that developed a prediction model for renal diseases examined general population^9, 18, 32^ or individuals with CKD^10–12, 14, 18, 22, 23, 26, 28, 31–34^. Only three prediction models were developed for patients with diabetes. The ADVANCE study enrolled 11,140 type 2 diabetes patients aged 55 years or older in 20 counties for 5 years. This study predicted two kidney-related outcomes, namely, doubling of serum creatinine to >= 2.26 mg/dL, renal replacement therapy, or renal death and new-onset albuminuria^15^. The TREAT study was a prospective cohort study nested within a randomized clinical trial to establish ESRD risk and composite of death or ESRD risk models using serum levels of the cardiac biomarkers TnT and NT-pro-BNP in 4038 patients with type 2 diabetes, anemia, and CKD^14^. The RENAAL study was also a prospective cohort study nested within a multinational randomized control trial to develop ESRD risk scores in 1,513 patients with type 2 diabetes and nephropathy^13^. The former two prediction models demonstrated good discrimination ability (C statistic of 0.84–0.85)^14, 15^, whereas the RENAAL study did not report statistics for discriminatory ability. The predictors identified in these three studies, such as age, creatinine, blood pressure, albuminuria, diabetic retinopathy, and insulin use, were similar to those in our model. In addition to these factors, our prediction model also considers HbA1c variation, one of key indicator for diabetes care. Besides blood pressure level, our prediction model integrates information of anti-hypertensive medication and variation in SBP. Our prediction model showed excellent discrimination ability (AUCs of 0.90). The frequency of 3-, 5- and 8-year observed and predicted events were similar in the derivation and validation sets and in the Hosmer–Lemeshow *x* ^*2*^ test. Our prediction models fit the data with good prediction ability.

Risk prediction models can provide individual estimates of risks to specific diseases and serve as guidance for the clinical management of high risk patients. The use of the risk scores for risk stratification can be helpful to clinicians or health policy providers to provide interventions such as diets to modify behaviors to reduce ESRD risk and health cost. It has been reported that dietary intervention improved renal function measured by eGFR in moderately obese persons with moderate impaired renal function^35^. Several disease risk models have been developed and validated in Taiwan; these models are targeted for diseases including stroke in general population^36^, type 2 diabetes^37^, cirrhosis and hepatocellular carcinoma^38–40^, gastric cancer^41^, and periodontal disease^42^. There was no risk prediction model being established to determine ESRD in patients with type 2 diabetes in Taiwan. The present study developed a risk score to predict the 3-, 5-, and 8-year ESRD risk in patients with type 2 diabetes in Taiwan.

### Strengths and limitations

Our study has the advantages of a relatively large group of patients with type 2 diabetes and a long-term follow-up period of 8.3 years. Our study took into account a wide range of potential risk factors. Baseline macro- and microvascular diseases were evaluated and chosen in our prediction model. Medical management for the control of diabetes, hypertension, and hyperlipidemia were also considered in our model. Baseline renal function, albuminuria, and visit-to-visit variation in HbA1c and SBP play principal roles in our prediction model. The present study showed excellent discrimination ability (AUCs of 0.90) for ESRD risk score in Chinese patients with type 2 diabetes.

Our research has five limitations. First, we obtained data on renal function, HbA1c-CV, SBP-CV, medication data, and risk factors from 2001 to 2003, but we did not had data after 2003. Exposure status may have changed during the follow-up period. In addition, the ESRD risk score was not able to predict ESRD risk longer than 8 years because of unavailability of data for ESRD. Second, the NDCMP database did not contain any physical activity, diet, genetic risks, and cardiac biomarkers that may be associated with ESRD risk. Third, although these patients were prospectively followed up by care managers, we had no control over the nature and the quality of the measurements that were made because the study design was retrospective. In addition, the diagnosis of comorbidity is based on ICD codes, which were dependent on the diagnostic accuracy of our database. The accuracy of diagnoses in the database had been improved by the routine check on samples of medical charts conducted by the bureau of NHI in Taiwan^43^. The punishment on every false diagnostic report was severe. Further, to ensure accurate diagnosis of comorbidity, we included only those cases with at least three outpatient visits or at least one hospitalization. Therefore, the prevalence of comorbidity could be somewhat underestimated. This kind of underestimation might be random. Because there is no evidence indicates that undiagnosed comorbidity is associated with ESRD. Thus, this error results in the effect may be toward the null, a lesser threat to validity. Fourth, our study had NDCMP participants as study subjects, which may result in selection bias. In order to assess the possibility of selection bias, we assessed age and gender distributions between NDCMP participants and population with type 2 diabetes in Taiwan. We found similar distributions and non-differential distributions in age and sex demonstrated this kind of selection error might be random, resulting in the effect toward the null, a lesser threat to results’ validity. Last, the primary outcome measure, ESRD, was ascertained by ICD-9-CM code in catastrophic illness certification from the Registry for Catastrophic Illness database of NHI program. Because each individual registered in the catastrophic illnesses database is exempted from any co-payment for treatment, the process for evaluating applicants’ eligibility for this registry is strict and comprehensive. For the case of ESRD, the catastrophic illness certification was issued by a nephrologist and confirmed by another nephrologist. Under this condition, the possibility of ESRD cases identified in this study being true positive are high and there is a likelihood of underestimation of ESRD incidence. If a true association exists between each factor and ESRD incidence, then this type of underestimation would result in reduction in the power of the study for detecting such an association. Even though the power of the present study may be lessened, the prediction model has very good predictive and discriminatory ability.

In conclusion, our study developed 3-, 5-, and 8-year ESRD risk scores with good prediction accuracy and discriminatory ability. Our study need additional sample for further external validation. Our results demonstrate the risk prediction model is a useful screening tool for preventing ESRD in Chinese patients with type 2 diabetes. This tool can guide clinicians in planning intervention and providing information for policy makers to set up public health strategies to prevent ESRD and reduce healthcare costs.

## Methods

### Data source

The National Diabetes Care Management Program (NDCMP), which was established by National Health Insurance (NHI) program in 2001, enrolled patients with type 1 and type 2 diabetes. NDCMP emphasized coordinated physician-led multidisciplinary team care. It enhanced high quality health care through increased monitor frequency, self-care education and annual diabetes-specific physical examinations and laboratory tests by provision of additional financial incentives. NDCMP required health professionals in fields of endocrinology, nephrology, internal medicine, cardiology, family medicine, and others to have clinical education and training programs to become certificated and eligible to be care providers of NDCMP, who voluntarily enroll patients into this program. Standardization of clinical practice, including care and treatment, assessment and diagnosis of diabetic complications were enhanced by these clinical continuing education and training programs. A retrospective cohort study was conducted in patients with type 2 diabetes who enrolled in the NDCMP from 2001–2004. Date of entry was defined as the index date. We used the NDCMP and National Health Insurance Research Database (NHIRD) to construct a cohort of patients with type 2 diabetes. We combined the datasets of NDCMP and NHIRD, including the details of ambulatory care orders of NHIRD from 2002–2004. Such approach enabled us to acquire information on baseline characteristics, including socio-demographic factors, duration of type 2 diabetes, age of onset, diabetes-related factor and biomarkers, comorbidity, types of anti-diabetes medications, hypertension medications, cardiovascular medications, and hyperlipidemia medications. We then used inpatient and outpatient databases of Taiwan NHIRD from 2001 to 2011 to obtain subsequent ESRD events 1 year after the index date to 2011. Each patient was followed up from the date of entry until 31 December 2011. Patients were also monitored for withdrawal from NHI program, death, or development of ESRD. We used baseline characteristics to build a point-based prediction model for ESRD risk in patients with type 2 diabetes.

### Study subjects

**Figure 3:**
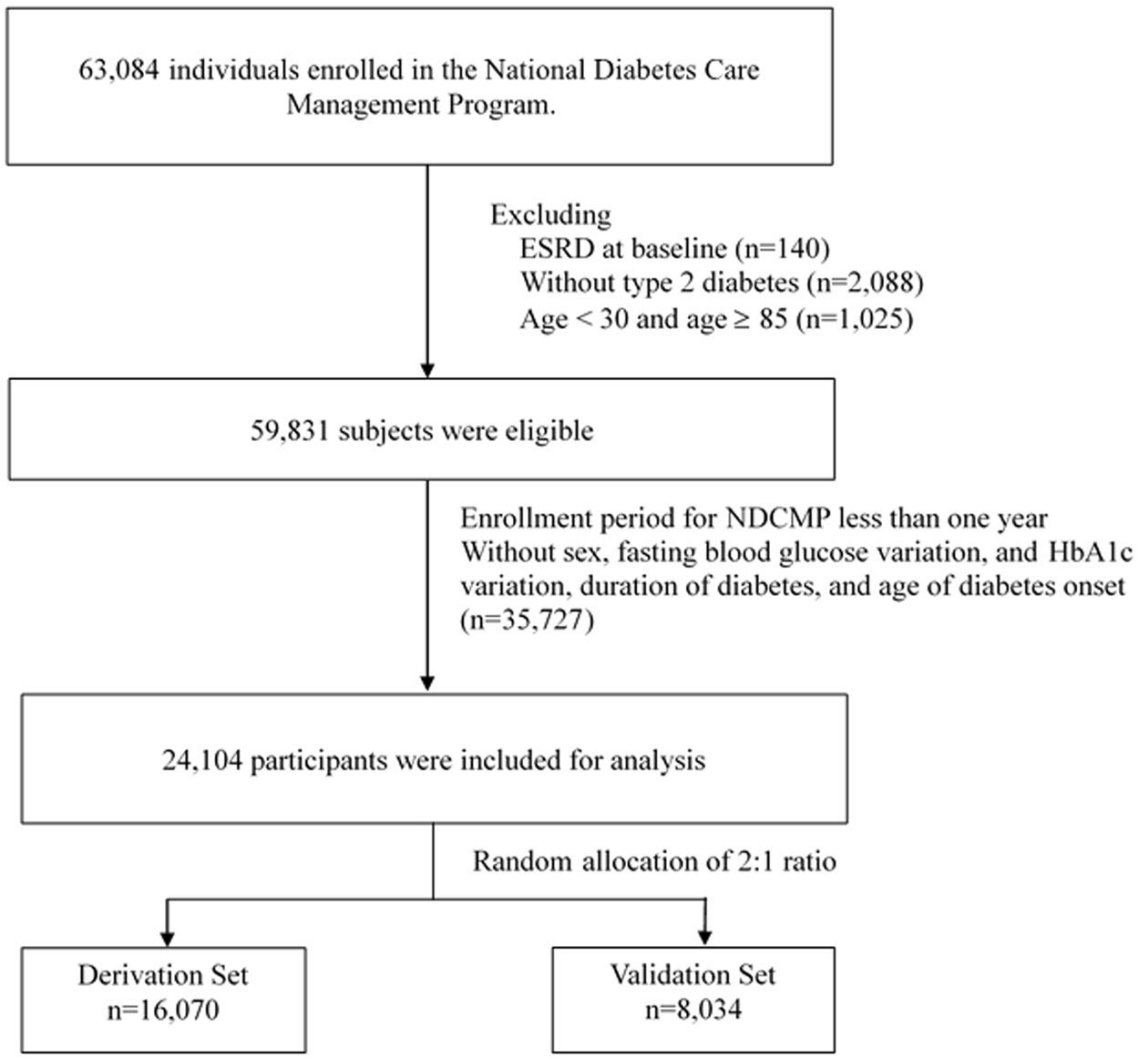
Flowchart for recruitment procedures of the predictive model for ESRD.

A total of 63,084 enrolled diabetic patients were diagnosed with type 2 diabetes based on the criteria of American Diabetes Association in the NDCMP from 2002 to 2004. We included patients who had at least 1 year of follow-up for calculation of visit-to-visit variation in glycated hemoglobin (HbA1c) and blood pressure, and without ESRD at baseline or missing information regarding baseline characteristics of comorbidities and laboratory blood test results. Figure 3 shows the flowchart of the recruitment procedure in the study. Baseline factors were com_pared between patients included and those excluded using standardized mean differences. We observed most of standardized mean differences were less than 0.1 standard deviations (SD), indicating a negligible difference in proportions or means between included and excluded patients. Although the standardized mean differences of fasting plasma glucose, eGFR, and variation in HbA1c were greater than 0.1 (0.11, 0.11, and 0.11, respectively), the mean values between excluded and included patients were acceptable (179.15 ± 11.471.6 vs. 171.67 ± 64.83 for fasting plasma glucose; 75.86 ± 23.12 vs. 73.38 ± 21.78 for eGFR; and 18.14 ± 16.88 vs. 16.47 ± 14.64 for variation in HbA1c). A total of 24,104 participants were randomly assigned to a derivation set (n = 16, 070) and a validation set (n = 8,034) at a 2:1 ratio. This study was approved by the Human Research Committee of China Medical University Hospital and all methods were performed in accordance with the relevant guidelines and regulations. Informed consent of the study participants was not required because the dataset used in this study consists of de-identified secondary data released for research purposes.

### Variables

Independent variables included sociodemographic factors, lifestyle behaviors such as smoking and alcohol drinking, diabetes-related factors and biomarkers, comorbidity, and medication use. Three types of personal information (sociodemographic factors, lifestyle behaviors, and diabetes related factors and biomarkers) were obtained at baseline from NDCMP database by case managers who did not know the objectives of the present study objective. The coefficient of variation (CV) for HbA1c and SBP measurements from outpatient visits within the first year of index date for each patient were calculated for those with more than two HbA1c and SBP measurements in the first year. To adjust for the possibility that the number of visits might affect variation, the CV of HbA1c and SBP was divided by square root of the ratio of total visits divided by total visits minus 1. Comorbidity and medication use of individual patients were retrieved from NHIRD database for 1-year period after index date. Comorbidity was determined by at least three service claims for outpatient care, or one service claim for inpatient care. The comorbidities of hypertension (ICD-9-CM codes 404–405), stroke (ICD-9-CM codes 431–438), cardiovascular disease (ICD-9 code 410 to 413, 414.01 to 414.05, 414.8 and 414.9), peripheral neuropathy (ICD-9-CM codes 356), diabetes retinopathy (ICD-9-CM codes 362.0), hypoglycemia (ICD-9-CM codes 250.3, 250.8, 251.0, 251.1, 251.2, 270.3, 775.0, 775.6, and 962.3), chronic kidney disease (ICD-9 codes 585), ketoacidosis (ICD-9-CM codes 250.1), postural hypotension (ICD-9-CM codes 458), arterial embolism and thrombosis (ICD-9-CM codes 444), hyperlipidemia (ICD-9-CM codes 272), and albuminuria (ICD-9-CM code 719.0 or urinary albumin-to-creatinine ratio ≥30 mg g^−1^ creatinine) from NDCMP were identified. History of medication included anti-diabetes medications (no medication, oral anti-diabetes drug (OAD), insulin monotherapy, and insulin plus OAD), hypertension medications (ACE inhibitors, ARBs, β-blockers, calcium channel blockers, and diuretics), cardiovascular medication (antiarrhythmic, anticoagulants, antiplatelet, digoxin, and nitrates) and hyperlipidemia medications (statins and fibrates).

The primary outcome was ESRD incidence, which was ascertained from catastrophic illness certification (ICD-9-CM code 585 with V45.1) database. In Taiwan, the diagnosis of ESRD is based on tests and exams including a discussion of health history, physical exam, blood and urine tests, and imaging test for assessment of kidney’s structure and size to look for abnormalities. The classification of chronic kidney disease adopts the National Kidney Foundation’s (NKF) Kidney Disease Outcomes Quality Initiative (KDOQI) staging criteria according to estimated glomerular filtration rate (eGFR) and the presence of kidney damage^44, 45^. The Modified Diet in Renal Disease (MDRD) formula is used as the equation for estimating GFR^44^. An individual is defined as ESRD case if his/her level of eGFR is less than 15 mL/min/1.73 m^2^, accompanying by signs and symptoms of uremia, or he/she needs for initiation of kidney replacement therapy (dialysis or transplantation) for treatment for complications of decreased GFR. We identified individuals who experienced ESRD from 1 year after their enrollment in NDCMP to 31 December 2011. This approach enabled the elimination of potential inverse causality. To enable patients with ESRD to obtain certificates from NHI and to be registered in the catastrophic illness certification database, they had to be diagnosed by at least two nephrologists who will assert their ESRD status. Given this requirement, patients who have been diagnosed with ESRD were less likely to be false positives. In addition, the validity of administrative claim data was assessed by the expert reviews performed by NHI Bureau on random samples of every 50 to 100 outpatient and inpatient claims in each hospital and clinic. Because the outcome ascertainment had been done in clinical setting, health professionals assessing the outcome status were not aware of the objective of the study, i.e., outcome assessment being blind.

### Statistical analysis

Proportions were presented for categorical variables. Means and standard deviations (SDs) were presented for continuous variables. The effect sizes were calculated to describe baseline characteristics of derivation and validation sets. The hazards ratios of predictor variables were estimated using Cox’s proportional hazards models to develop a prediction model of ESRD in the derivation set and to assess the model’s predictive accuracy in the validation set. We have four steps to select independent variables that result in a “best” model^46^. First, we conducted a careful univariable analysis of each variable. Second, we selected variables with univariable test of a *p*-value < 0.25^47, 48^ as a candidate for our multivariable model. Third, we constructed a multivariable model with candidate variables without collinearity. In addition, only variables with p-value < 0.05 were retained. Lastly, after refining a main effects model, we checked assumption of Cox’s proportional hazard model for all variables in our multivariate model. We verified the proportional hazards assumption by the graph of the log (−log(survival)) versus the log of the survival time graph and by comparing the regression coefficients from models censored at 3, 5, and 8 years.

The steps for predictive model development were based on Framingham Heart study in the determination of the ESRD risk score^49^. The seven steps were as follows: (1) estimating the parameters of the multivariable Cox’s proportional hazards model with backward elimination approach for model building strategy; (2) grouping the risk factors into categories and determine their reference values *W*_*ij*_; (3) assigning a score for each category to determine the referent risk factor profile; a base category for each risk factor has a 0 score; (4) determining the distance from the base category to each category in regression units; (5) setting the constant B, which is the number of regression units that reflect 1 point in the final points system; (6) calculating the number of points for each category of each risk factor, where Point_ij_ = (*W*_ij_ − *W*_*iREF*_)/*B*; and (7) determining the prediction risks for all possible total scores. The risk of ESRD was calculated by the following equation: $\hat{p}=1-{S}_{0}{(t)}^{\exp ({\rm{\Sigma }}{\beta }_{i}\times {X}_{i}-{\beta }_{i}\times {\bar{X}}_{i})}$, where $\hat{p}$. is the baseline disease-free probability, β_*i*_ is the regression coefficient for *X*_*i*_, and the ${\bar{X}}_{i}$ is the mean level of *X*_*i*_. The calculation of constant B depends on age considered in multivariate model. If we consider eGFR as a risk factor in the prediction model, it will result in collinearity issue due to overlapped information of age and eGFR. Thus, serum creatinine is considered in our study instead of eGFR. There were six continuous variables being categorized in step 2. Age was classified into categories by 5 years for an interval, the same as Framingham study; variations in HbA1c and systolic blood pressure were based on their tertiles; total cholesterol was based on NCEP ATP III criteria; systolic and diastolic blood pressure were based on classification of blood pressure for WHO/ISH reports^50^; serum creatinine was according to proposed classification/staging system for acute kidney injury based on modification of RIFLE criteria^51^. The receiver operating characteristic (ROC) curve analysis was applied to assess the predictive accuracy, and area under curve (AUC) was used to assess the discriminatory ability of the predictive model. Goodness-of-fit was performed by comparing the observed and predicted events of ESRD based on risk groups of deciles using the Hosmer–Lemeshow *x* ^2^ test. Internal validation was carried out to correct the potential for overfitting or “optimism” by using 1000 times bootstrap resampling^52^. Model calibration was conducted to evaluate the agreement between model-predicted probabilities and observed probabilities. We used calibration-in-large approach to calculate the intercept for evaluation of the extent whether predictions are systematically too low or too high. When the value of intercept is close to zero, it indicates that there is no systematic deviation of estimation of predicted probabilities. In addition, we calculate the calibration slope, an estimate of extremeness of predicted probabilities to test whether its value deviates from the ideal of 1.0. If the value of slope is close to one, it would reflect there is no overfitting of a model, i.e., there is no condition that the probability of an ESRD event tends to be underestimated in low risk patients and overestimated in high risk patients. Furthermore, the mean absolute error in calibration for slope and intercept were reported for assessing calibration, with error referring to the difference between the observed values and the bias-corrected calibrated values. We used a multiple imputation method to impute missing data for sensitivity analysis. A total of 28,424 subjects were imputed for missing data of variation in HbA1c and systolic blood pressure because of too short duration to calculate these two measurements. The method we used is multiple imputation method with a fully conditional specification (FCS) method, assuming the existence of a joint distribution for baseline variables of age, age of diabetes onset, duration of type 2 diabetes, and variation in HbA1c and systolic blood pressure, and regression analysis as imputation method. We performed statistical analysis using SAS version 9.4 (SAS Institute Inc., Cary, NC). Two-tailed p < 0.05 denotes statistical significance.

## Electronic supplementary material

Supplementary information
