# Supplementary material for: Development and validation of a risk prediction model for end-stage renal disease in patients with type 2 diabetes
Source: Sci Rep. 2017 Aug 31;7:10177. doi: 10.1038/s41598-017-09243-9 (PMC5579050; doi:10.1038/s41598-017-09243-9)
Supplement: Supplementary file 1 — Supplementary information [file 41598_2017_9243_MOESM1_ESM.pdf]

Supplementary information

Development and validation of a risk prediction model for end-stage renal disease in patients with type 2 diabetes

Cheng-Chieh Lin,†[1,2,3] Chia-Ing Li,†[1,3] Chiu-Shong Liu,[1,2,3] Wen-Yuan Lin,[1,2] Chih-Hsueh Lin,[1,2] Sing-Yu Yang,[4] Tsai-Chung Li[4,5*]

1. School of Medicine, College of Medicine, China Medical University, Taichung, Taiwan
2. Department of Family Medicine, China Medical University Hospital, Taichung, Taiwan
3. Department of Medical Research, China Medical University Hospital, Taichung, Taiwan
4. School of Public Health, College of Public Health, China Medical University, Taichung, Taiwan
5. Department of Healthcare Administration, College of Medical and Health Science, Asia University, Taichung, Taiwan

† Equally contributed as first author

* Corresponding authors: Tsai-Chung Li, China Medical University, 91 Hsueh-Shih Road, Taichung, 40421, Taiwan, Tel: 886-4-22052121ext. 6605, Fax: 886-4-22060248, e-mail: tcli@mail.cmu.edu.tw

Supplementary Table 1. Baseline characteristic of the study population in excluded group and included group

Variables	Excluded group (n=35,727) MEAN±SD* or n (%)	Included group (n=24,104) MEAN±SD* or n (%)	Standardized effect size
Socio-demographic factors			
Age (years)	60.13±11.42	61.09±10.76	-0.09
Gender			-0.02
Female	18553 (52.00)	12815 (53.17)	
Male	17128 (48.00)	11289 (46.83)	
Smoking habit	5734 (16.05)	3777 (15.67)	0.01
Alcohol drinking	2985 (8.36)	2186 (9.07)	-0.03
Age of diabetes onset (years)	54.37±11.15	54.44±10.83	-0.01
Duration of type 2 diabetes (years)	6.42±6.75	6.68±6.35	-0.04
Body mass index (kg/m^2)	25.56±4.00	25.71±3.75	-0.04
Obesity	12224 (34.22)	8854 (36.73)	-0.05
Diabetes-related factor and biomarker			
Systolic blood pressure (mm Hg)	134.58±18.43	134.86±17.72	-0.02
Diastolic blood pressure (mm Hg)	80.17±11.09	80.04±10.5	0.01
HbA1c level (%)	8.39±2.07	8.2±1.93	0.09
Fasting blood glucose (mg/dL)	179.15±71.6	171.67±64.83	0.11
Low-density lipoprotein (mg/dL)	118.08±31.43	117.86±31.27	0.01
High-density lipoprotein (mg/dL)	46.26±14.25	46.28±13.99	0.00
Creatinine (mg/dL)	1.06±0.63	1.05±0.53	0.02
Serum glutamate-pyruvate transaminase (u/l)	32.69±34.97	32.19±32.73	0.01
Total cholesterol (mg/dL)	198.85±47.60	195.72±42.04	0.07
Triglyceride (mg/dL)	181.43±146.12	171.92±130.92	0.07
eGFR (mg/dL)	75.86±23.12	73.38±21.78	0.11
Variation in fasting blood glucose (%)	34.28±28.60	32.01±25.67	0.09
Variation in HBA1c (%)	18.14±16.88	16.47±14.64	0.11
Variation in systolic blood pressure (%)	7.70±6.06	7.39±5.55	0.06
Variation in diastolic blood pressure (%)	7.92±6.61	7.71±6.1	0.03
Comorbidity			
Hypertension	15023 (42.05)	11031 (45.76)	-0.07
Stroke	2060 (5.77)	1176 (4.88)	0.04
Cardiovascular disease	3022 (8.46)	2150 (8.92)	-0.02
Peripheral Neuropathy	164 (0.46)	220 (0.91)	-0.06

Diabetes retinopathy	543 (1.52)	324 (1.34)	0.02
Disease of peripheral circulatory disturbance	1441 (4.03)	955 (3.96)	0.00
Hypoglycemia	1383 (3.87)	1016 (4.22)	-0.02
Chronic kidney disease	375 (1.05)	232 (0.96)	0.01
Ketoacidosis	355 (0.99)	196 (0.81)	0.02
Postural hypotension	25 (0.07)	21 (0.09)	-0.01
Arterial embolism and thrombosis	69 (0.19)	38 (0.16)	0.01
Peripheral vascular disease	214 (0.6)	208 (0.86)	-0.03
Hyperlipidemia	8207 (22.97)	6394 (26.53)	-0.08
Outcome			
ESRD	2126 (5.95)	1215 (5.04)	0.04

*: SD = standard deviation

Supplementary Table 2. ESRD risk score based on the final multivariate Cox's proportional hazards model with multiple imputation dataset (n=52,528) and complete dataset (n=24,104)

Risk factor	β (SE)<br>Dataset with multiple imputation	β (SE)<br>Complete dataset
Socio-demographic factors		
Age	0.01 (0.002)	0.02 (0.004)
Gender (male)	0.03 (0.04)	-0.01 (0.07)
Age of diabetes onset (years) (ref: <45)		
≥45	-0.62 (0.05)	-0.56 (0.10)
Diabetes-related factor and biomarker		
Creatinine (mg/dL) (ref: <2.0)		
2.0–4.0	2.59 (0.05)	2.57 (0.10)
>4.0	3.10 (0.10)	2.65 (0.22)
Variation in HbA1c (%) (ref: <8.5)		
8.5–17.5	0.06 (0.05)	0.23 (0.09)
>17.5	0.26 (0.05)	0.48 (0.09)
Variation in systolic blood pressure (%) (ref: <4.4)		
4.4-8.7	-0.06 (0.05)	-0.08 (0.09)
>8.7	0.04 (0.05)	0.24 (0.09)
Diabetes-related disorders		
Diabetes retinopathy	1.18 (0.08)	0.88 (0.17)
Albuminuria	0.40 (0.06)	0.50 (0.09)
Medication use		
Anti-diabetes medications (ref: no medication)	ref	ref
Oral only	-0.57 (0.07)	-0.60 (0.21)
Insulin	0.33 (0.09)	0.45 (0.23)
Insulin+oral agent	0.15 (0.09)	0.12 (0.23)
Hypertension medications		
No		
SBP<130 and DBP<85	ref	ref
SBP:130-139 or DBP:85-89	0.02 (0.10)	0.12 (0.21)
SBP:140-159 or DBP:90-99	0.37 (0.09)	0.46 (0.20)
SBP≥160 or DBP≥100	0.89 (0.10)	0.72 (0.25)
Yes		
SBP<130 and DBP<85	0.73 (0.09)	0.99 (0.17)
SBP:130-139 or DBP:85-89	0.79 (0.08)	1.06 (0.17)
SBP:140-159 or DBP:90-99	1.13 (0.08)	1.33 (0.16)
SBP≥160 or DBP≥100	1.46 (0.08)	1.65 (0.17)
Hyperlipidemia medications		
No		
Total cholesterol: <200	ref	ref
Total cholesterol: 200-239	0.21 (0.06)	0.34 (0.12)

Total cholesterol: ≥240	0.71 (0.07)	0.53 (0.17)
Yes		
Total cholesterol: <200	0.29 (0.06)	0.36 (0.11)
Total cholesterol: 200-239	0.31 (0.06)	0.35 (0.11)
Total cholesterol: ≥240	0.89 (0.06)	0.91 (0.11)

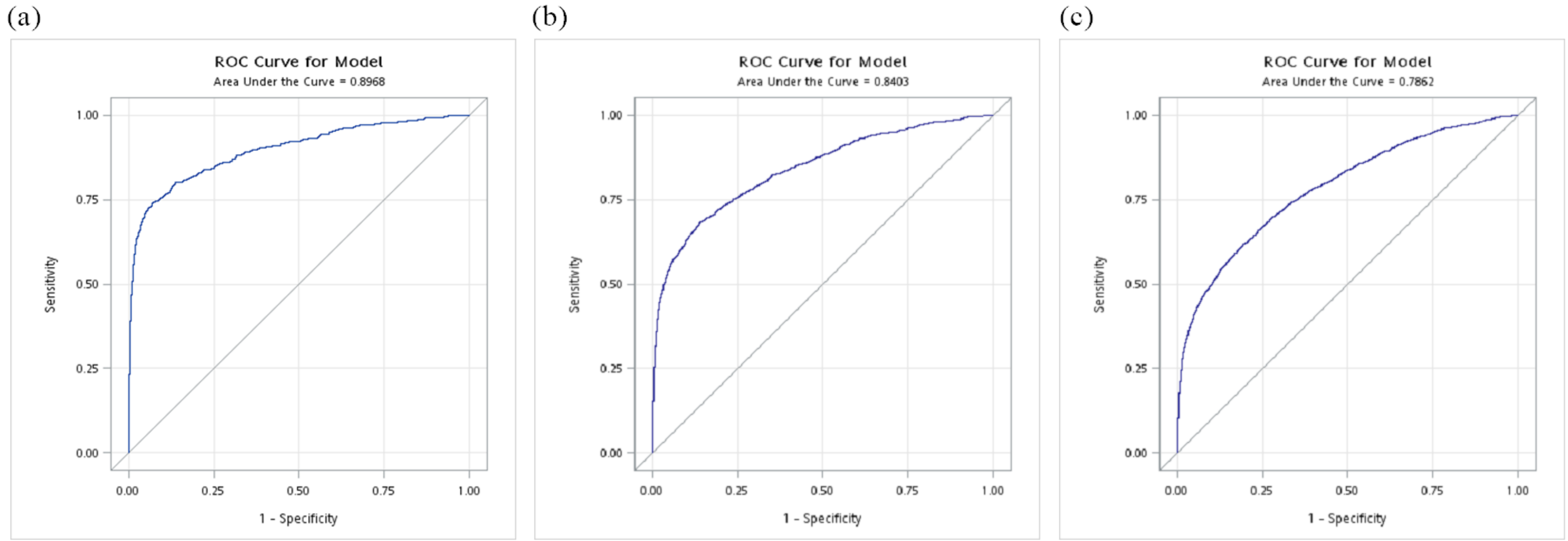

Supplementary Figure 1. Receiver-operating characteristiccurve (ROC) for (a) 3-year (b) 5-year (c) 8-year ESRD riskin sensitivity analysis (N=52,528)

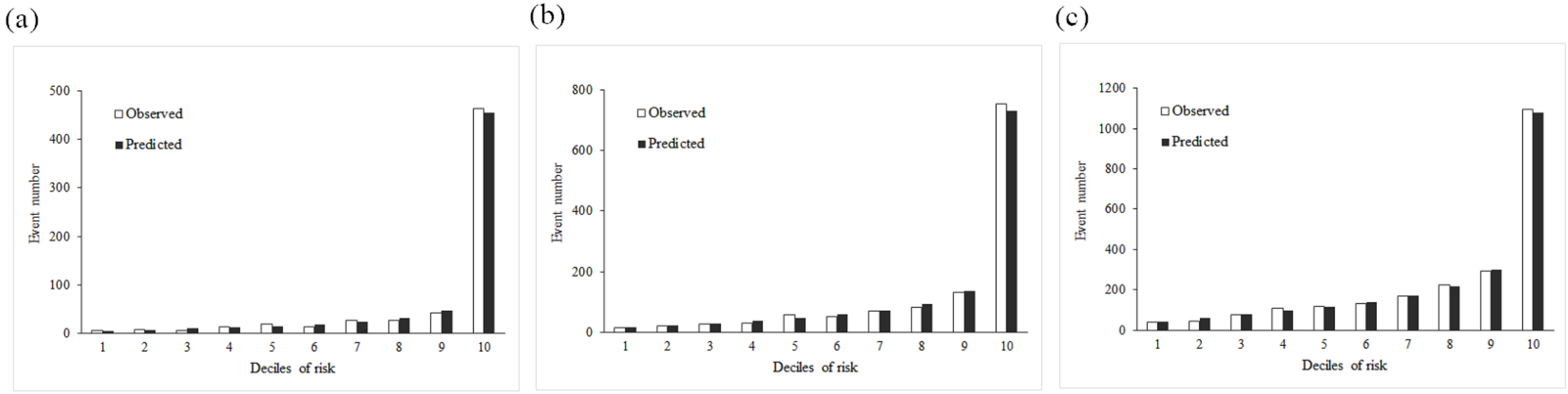

Supplementary Figure 2. Predicted versus observed ESRD numbers according to deciles of (a) 3-year (b) 5-year and (c) 8-year risk in sensitivity analysis (N=52,528)

(a)

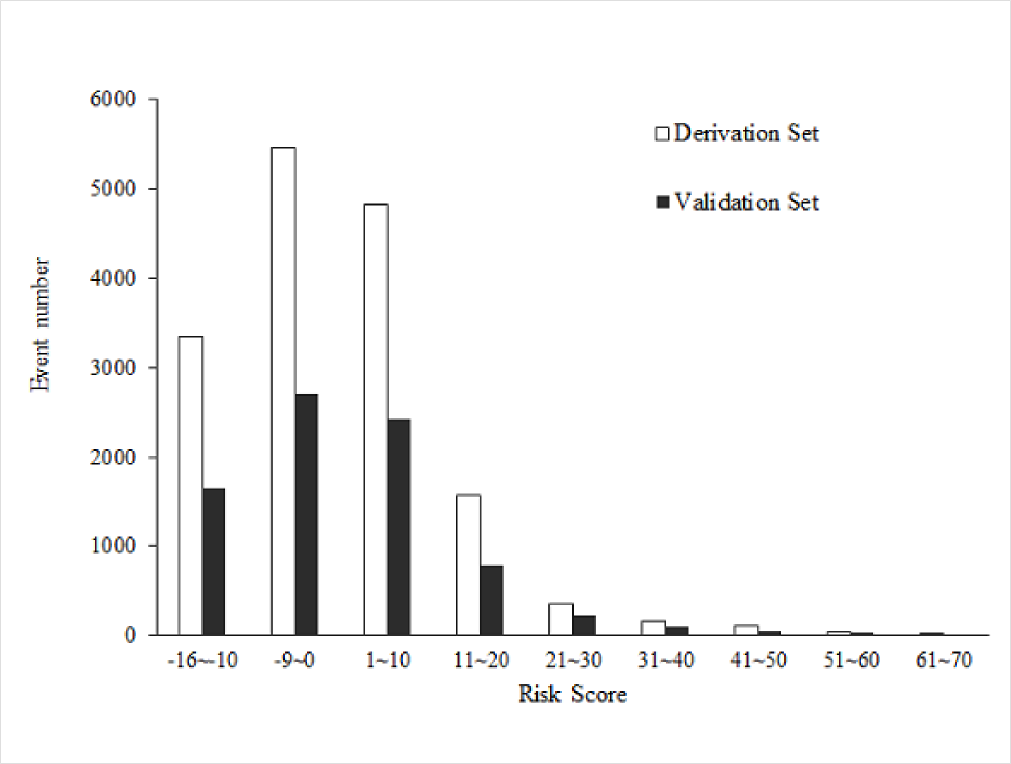

(b)

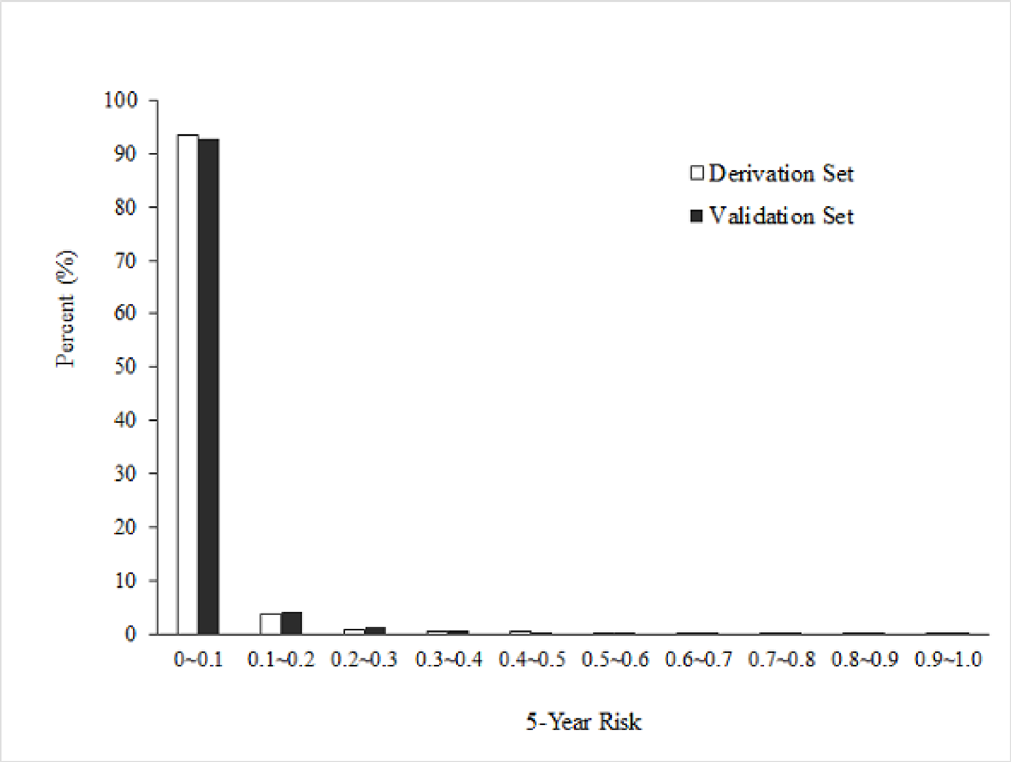

Supplementary Figure 3. The distributions of risk score (a) and 5-year risk (b) for derivation and validation sets
